# Editorial: The Recovery College model: state of the art, current research developments and future directions

**DOI:** 10.3389/fpsyt.2025.1745050

**Published:** 2025-12-02

**Authors:** Catherine Briand, Martine Vallarino, Filippo Rapisarda, Anne Schanche Selbekk

**Affiliations:** 1 Department of Occupational Therapy, University of Quebec at Trois-Rivières, Trois-Rivières, QC, Canada; 2 Research Center of Institut universitaire en santé mentale de Montréal, Montréal, QC, Canada; 3 Department of Public Health, The Faculty of Health Sciences, University of Stavanger, Stavanger, Norway

**Keywords:** Recovery College model, state of the art, research direction, mental health service and education, co-production and co-learning, recovery, mental health, learning environment

## Introduction

Established in England in 2009, Recovery Colleges (RCs) are educational hubs offering free, co-produced courses on mental health, well-being, and collective living (1, 2). According to Hayes (3), there are 221 RCs across 28 countries on five continents. Rooted in the principles of mutual learning, inclusivity, and respect for diversity, RCs bring together individuals with lived experience of mental illness, health practitioners, and community members in a co-learning space where experiential, clinical, and theoretical knowledge are considered complementary and equally valuable.

The distinctive feature of the RC model is its value-driven, non-hierarchical approach to diverse knowledge. Rather than focusing on symptom reduction, RCs aim to foster recovery through empowerment, self-determination, and co-production within transformative, anti-stigma learning environments.

This Research Topic offers an international overview of the current state of the RC model. Research teams from the UK, Canada, Norway, Sweden, the Netherlands, and Italy have contributed to this Research Topic. Their articles examine a wide range of issues, including active ingredients and mechanisms of action, specific outcomes, implementation challenges, and strategies to ensure quality and fidelity, highlighting the richness and complexity of RC practices from an international perspective.

## Contents of the Research Topic

This Research Topic brings together 10 contributions that explore the RC model through various lenses, offering an in-depth and multifaceted understanding of its evolution, implementation, and impact.

The first article, written by the authors of this editorial, provided a state-of-the-art review of the studies published since the initial RC model studies. Briand et al. conducted a comprehensive systematic review of RC evaluative studies published between 2013 and 2024. Analysis of 64 articles revealed five qualitative clusters. Early articles on RCs focused on implementation stages and lessons learned (2013-2024). Next, articles focused on perceived benefits, learners’ experiences, and active ingredients (2014-2024). Articles then moved on to outcome evaluation (2015-2024) and service utilization and costs (2019-2024). Finally, articles focused on documenting an international scope of RCs, providing a status report, and global multicenter comparisons (2019-2023). These qualitative clusters capture not only the scope and richness of the studies, but also the progression in study quality over the past 10 years. To keep pace with this progression, future studies need to consolidate outcome measurements, increase international and multicenter studies, and more systematically measure the quality of implementation and the support needed for trainers to ensure this quality. The articles presented in this Research Topic provide some answers to these challenges. The articles can be grouped into three thematic groups: (1) understanding the learning framework, (2) implementation recommendations, and (3) measuring outcomes.

The first group of articles focused on understanding how the RC learning framework functions and how it drives change. RCs offer a unique social space that requires the embodiment of values through concrete principles and operations. This learning space is complex and fragile. The three articles in this group discussed this topic in great depth, providing an even better understanding of the RC model.

- Lefay et al. proposed a hermeneutic analysis of the RC learning model, highlighting connections with the key learning theories. According to their study, the RC model integrates important concepts from social constructivism, cognitive constructivism, andragogy, and transformative learning. Through an analysis of the founding texts, this article reviews the mechanisms of action, principles, and operations of the RC model, along with the role of RCs in addressing epistemic justice, power relations, and inclusive learning spaces.
- Then, Sjursæther et al., drawing on ethnographic research in two Norwegian RCs, examined how the social frameworks inhibit and promote sharing between learners and trainers within RCs. The authors identified five critical frames within the social framework: learning, recovery, strength-based, equality, and openness.
- Van Wezel et al. described the experience of an RC in the Netherlands, examining how peer support values are implemented and how participants experience this value-driven practice. The results highlight how RCs facilitate opportunities for recovery by fostering spaces for dialogue and co-creation, while revealing the fragility and the complexity of these spaces. Understanding their value requires examining how and when these spaces emerge or become constrained, in addition to the factors that influence these dynamics.

The second group of articles examined the conditions favorable to implementation and how we can better meet the needs of learners and better support and engage trainers. The complex implementation of the RC model requires continuous questioning of how to respect its core values and principles, adjust to its environment and needs, operate in an integrated way within the system, and achieve its goals (Parsons’ social action model). The four articles in this group provided a stimulating starting point for further reflection and development regarding course content selection, involvement of learners in course co-production, and better support for trainers.

- Takhi et al. conducted a large-scale document analysis of 2,300 courses’ documents relating to 71 RCs in the England to develop a typology of RC courses and assess the differences between course types across RC orientations. Their findings suggest that RCs support mental health recovery through a diversified curriculum of courses. This work classified courses into 14 superordinate categories; the three most common ones were Self-Management of Well-being, Mental Health Conditions and Symptoms, and Creativity. However, more courses for family carers need to be developed. The article described next will address this issue.
- Bowness et al. conducted a participatory action research project involving family carers as learners. Family carers helped co-produce and co-evaluate the course to ensure that it met their needs. This example shows how participatory action research can be an effective approach to designing courses for family carers in an RC.
- As part of a participatory action research approach conducted in Canada, Parsa et al. explored key factors and best practices for supporting peer trainers (trainers with experiential knowledge) in their engagement and retention within RCs. The authors identified five themes to support peer trainers’ engagement: inclusivity, connectivity, adaptability, empowerment, and implementation factors. Practical recommendations were made regarding recruitment, training, and workplace support.
- Vallarino et al. conducted a qualitative multicenter study that explored the experiences of RC trainers in Quebec (Canada) and Lombardy (Italy) who work in complementarity of knowledge. The authors identified eight main themes: the distinctive nature of the RC model, the development of core competencies, the dynamics within the trainers’ dyads, the strengths and challenges of the co-production process within the dyad and with learners, the ongoing activities and tools to ensure trainers’ alignment with the model, and the activities to support the trainer’s role. The results suggest the importance of raising awareness among trainers about the relevant elements to be considered when designing and implementing an RC training program.

The third group of articles focused on measuring and understanding outcomes. In recent years, RC courses have addressed the needs of a wide variety of learners (e.g., youth, seniors, homeless people, health and educational professionals, and so on). Measuring outcomes must account for the specific effects on these diverse clienteles. The two articles in this group suggested new methodological avenues for future research.

- Alam et al. conducted a scoping review of potential outcome measures to assess the impact of RC courses on dementia. The lack of validated outcome measures in this context makes it difficult to evaluate the effectiveness of RC courses. Fourteen instruments related to hope, resilience, self-efficacy, empowerment, and adaptation were identified. However, the authors called for the development of more context-sensitive, relational, and recovery-oriented tools tailored to these specific populations.
- Rapisarda et al. applied cluster analysis techniques to repeated outcome variables collected from 353 learners in an online RC in Quebec, Canada. Their findings indicate that change trajectories follow three general patterns. Cluster A showed sustained gains in well-being and anxiety reduction, with moderate improvements in empowerment and resilience, but little change in stigma. Cluster B had moderate improvements in well-being and empowerment, slight stigma reduction, and no significant changes in anxiety or resilience, which is often seen among practitioners. Cluster C demonstrated progressive improvements in empowerment and anxiety reduction, slight resilience gains, initial stigma reduction, and stable well-being, a pattern commonly seen among service users.

## Conclusion

The authors who contributed to this Research Topic reached insightful conclusions that expanded knowledge regarding the state of the art of RC research. As highlighted in the systematic review by Briand et al., the field is progressing toward greater methodological rigor. We must continue in this direction.

Five future directions emerge clearly:

1. Strengthening outcome research with larger, more robust designs, long-term follow-up, and rigorous evaluation frameworks.
2. Expanding multicentric and international studies to reflect diverse settings and cultural dynamics.
3. Assessing outcomes and model fidelity in specific populations and contexts, including underrepresented groups such as LGBTQ+ individuals, vulnerable groups, older adults, and people living with cognitive impairments.
4. Clarifying and protecting model fidelity, while allowing for flexible, locally grounded adaptations that preserve RC values.
5. Embedding RCs into broader mental health strategies, including the delivery of training programs, stigma reduction, and community-based innovations.

Recovery Colleges have demonstrated the potential to foster personal empowerment, systemic change, and inclusive citizenship. Fully realizing this potential will require research that is both methodologically rigorous and grounded in lived experience—research that pays attention to context, egalitarianism, and the voices of those who are most often excluded. This Research Topic invites continued collaboration across disciplines, contexts, and countries.
